# Huntingtons Disease Mice Infected with *Toxoplasma gondii* Demonstrate Early Kynurenine Pathway Activation, Altered CD8+ T-Cell Responses, and Premature Mortality

**DOI:** 10.1371/journal.pone.0162404

**Published:** 2016-09-09

**Authors:** David W. Donley, Andrew R. Olson, Merl F. Raisbeck, Jonathan H. Fox, Jason P. Gigley

**Affiliations:** 1 Department of Veterinary Sciences, University of Wyoming, Laramie, WY, United States of America; 2 Neuroscience Graduate Program, University of Wyoming, Laramie, WY, United States of America; 3 Department of Psychology, University of Wyoming, Laramie, WY, United States of America; 4 Department of Molecular Biology, University of Wyoming, Laramie, WY, 82071, United States of America; University of Wisconsin Medical School, UNITED STATES

## Abstract

Huntington's disease (HD) is a progressive neurodegenerative disorder caused by a polyglutamine-repeat expansion in the huntingtin protein. Activation of the kynurenine pathway of tryptophan degradation is implicated in the pathogenesis of HD. Indoleamine-2,3-dioxygenase (IDO) catalyzes the oxidation of tryptophan to kynurenine, the first step in this pathway. The prevalent, neuroinvasive protozoal pathogen *Toxoplasma gondii* (*T*. *gondii*) results in clinically silent life-long infection in immune-competent individuals. *T*. *gondii* infection results in activation of IDO which provides some protection against the parasite by depleting tryptophan which the parasite cannot synthesize. The kynurenine pathway may therefore represent a point of synergism between HD and *T*. *gondii* infection. We show here that IDO activity is elevated at least four-fold in frontal cortex and striata of non-infected N171-82Q HD mice at 14-weeks corresponding to early–advanced HD. *T*. *gondii* infection at 5 weeks resulted in elevation of cortical IDO activity in HD mice. HD-infected mice died significantly earlier than wild-type infected and HD control mice. Prior to death, infected HD mice demonstrated decreased CD8+ T-lymphocyte proliferation in brain and spleen compared to wild-type infected mice. We demonstrate for the first time that HD mice have an altered response to an infectious agent that is characterized by premature mortality, altered immune responses and early activation of IDO. Findings are relevant to understanding how *T*. *gondii* infection may interact with pathways mediating neurodegeneration in HD.

## Introduction

Huntington’s disease (HD) is an autosomal dominant neurodegenerative disorder caused by a CAG-repeat expansion in exon-1 of the huntingtin gene (*HTT*) that results in expression of a polyglutamine-expanded huntingtin protein [1]. HD patients develop progressive motor, cognitive and psychiatric deterioration that results from degeneration of cortico-striatal-thalamic circuits. Mutant huntingtin protein (mhtt) expression results in disruption of many down-stream processes including energy metabolism, gene transcription and immune activation [2–4]. While CAG expansion size is the main determinant of age of disease onset there is considerable variation in age of onset after correction for CAG mutation size. This variability is explained in part by as yet unknown environmental modifiers of HD [5].

Mutant *HTT* gene-positive individuals demonstrate changes in properties of both peripheral and brain immune cells. Microglial activation, a marker of neuroinflammation, is present pre-symptomatically in striatum and cerebral cortex and progresses with HD pathology grade [6, 7]. Mutant huntingtin is expressed in peripheral immune cells including monocytes and T and B lymphocytes. Further, mhtt levels in these cells associates positively with disease burden and striatal atrophy in human HD [8]. Human HD monocytes demonstrate hyper-responsive pro-inflammatory cytokine production in response to lipopolysaccharide which stimulates innate immune response [9]. Further, studies in human HD have demonstrated pre-clinical elevations of serum pro-inflammatory cytokines [4, 10]. Studies in genetic mouse models of HD largely recapitulate findings in the human disease [11]. Together these studies support early alteration of both peripheral and brain immune cell function in HD.

*Toxoplasma gondii* (*T*. *gondii*) is a protozoan parasite with world-wide human prevalence of ~30% and results in clinically silent (latent) and life-long infection of brain in immune-competent individuals [12, 13]. Control of this pathogen depends upon interferon γ (IFNγ) produced by a number of cell types including CD8+ T-cells [14]. *T*. *gondii* is dependent on host-derived tryptophan for growth and survival and depletion of this amino acid is an important component of the protective immunity against *T*. *gondii* infection [15, 16]. Tryptophan breakdown involves activation of indoleamine-2,3-dioxygenase (IDO) catalyzing oxidation of tryptophan to kynurenine and represents the first step in the kynurenine pathway (KP) [17]. In brain, IDO is expressed in microglia. Importantly, activation of the KP generates intermediates that are implicated in the progression of HD [18, 19]. Additionally, peripheral inhibition of the kynurenine pathway may provide protection in HD mice [20]. Therefore, *T*. *gondii* upregulates an immune defense pathway that is implicated in the progression of HD suggesting that the two disease processes may interact.

Despite growing evidence for altered immune cell responses in HD, studies addressing the effects of infectious processes in HD have, to our knowledge, not been reported. The goal of the current study was to determine how HD mice respond to *T*. *gondii* with particular emphasis on IDO as a marker of KP activation and HD-associated neuroinflammation, and CD8+ T-cell phenotypic responses because of their critical importance for immune control of the parasite. We report that infection of N171-82Q HD mice results in premature mortality, early cortical IDO activation, and altered CD8+ T-cell responses. Findings indicate the potential for *T*. *gondii* infection to activate a pathway linked with HD progression.

## Materials and Methods

### Mouse husbandry and breeding

N171-82Q Huntington’s disease (HD) mice (Jackson Labs, Bar Harbor, ME; strain B6C3-Tg(HD82Gln)81Gschi/J) were maintained as described [21]. Mice were housed in OptiMice cages (Animal Care Systems) with 4–5 mice per cage and one genotype/treatment per cage. Sentinel mice are evaluated every 6 months by comprehensive serology panel screening for murine infectious diseases at Charles River Laboratories and are free of all tested diseases. Mice were humanely sacrificed using B-euthanasia solution or CO_2_ asphyxiation. This study and all procedures used were approved by the University of Wyoming Institutional Animal Care and Use Committee (UW-IACUC) (protocol numbers 20130911JG00019, 20150929JG00198, 20151106JG00206-01 and 20160301JF00214-BC) in accordance with the National Institutes of Health guidelines.

### Animal Model

The N171-82Q mouse is a transgenic model of HD that expresses the N-terminal 171 amino acids of human huntingtin protein with 82 poly-glutamine repeats under the control of the prion protein promotor [22]. These mice display a progressive phenotype with motor deficit and weight loss beginning around 8–9 weeks of age. They develop striatal and neocortical degeneration, as occurs in in human HD. Spontaneous mortalities begin at 14–15 weeks of age in this colony [21].

### Experimental Design

Female N171-82Q HD mice were used for all experiments. HD and wild-type litter-mate mice were weaned at 3.5 weeks of age and simultaneously, systematically assigned to experimental groups to balance ages and minimize effects of litter of origin. HD and wild-type mice were dosed with 100 bradyzoite-stage parasites or phosphate-buffered saline (PBS) at 5–6 weeks of age by intra-peritoneal injection. Experiments were a 2x2 factorial design. For all studies involving behavior or phenotypic analyses, the investigator was blinded to the treatment group. Another investigator coded cage cards so that mouse genotype and treatments were unknown. Individual mice were identified by a 3-digit identifier assigned during genotyping. This identifier was used to track individual mice throughout the study. Data remained blinded until just before data analysis. Mice were observed at least once daily and rated on a 5-point scale for clinical signs of *T*. *gondii* infection and overall sickness where 0 is normal appearance and 5 being non-ambulatory. Per the study approved by the UW-IACUC with an initial study endpoint of 16-weeks of age, mice that were non-ambulatory, unable to reach food or water, or were rated as a 4 for three consecutive days were humanely sacrificed. The observational health scale and these criteria were used to determine humane end points for euthanasia for the entire study period. Thirteen mice that were euthanized and forty-two mice that died unexpectedly. Two of the unexpected deaths were attributed to HD and nine were related to *T*. *gondii* infection. The cause of death was unclear for thirty-one mice. Early death of infected HD mice was unexpected in initial experiments; however, we had approval for survival as an outcome in UW-IACUC-approved studies. Experiments were terminated at ~14 weeks of age at which all remaining mice were humanely sacrificed. When statistical analysis of survival data was completed, mice sacrificed at the study endpoint were considered as censored values. Further experiments were designed to study mice before death occurs.

### Cyst-free *T*. *gondii* parasite preparation

The Me49 strain of *T*. *gondii* was harvested from young C57BL/6 or B6/C3H mice infected with 10 cysts by oral gavage and sacrificed 4–5 weeks post infection. Cyst-free parasites were prepared from these mice. In brief, freshly-collected brain tissue was homogenized on ice in 2 mls sterile phosphate-buffered saline (PBS) with a Dounce homogenizer for one minute. The brain homogenate was centrifuged at 2000xg for 5 minutes at 4°C and the supernatant discarded. The pellet was then resuspended in 20 mls of sterile 30% Percoll®. The mixture was centrifuged for 20 minutes at 2000 g and 15°C with no brake. Supernatant was carefully removed and the pellet resuspended in 100 μl of 0.25% (v/v) trypsin for 30 seconds and then diluted with 900 μl of PBS. The mixture was added to 4 mls of PBS and filtered through a 3 μm filter. The parasite concentration was estimated using a hemocytometer. Parasites were then diluted to 500 per ml in cold PBS. One-hundred parasites (0.2 mls) were given to each mouse by intra-peritoneal injection.

### Quantitative PCR for *T*. *gondii* burden in mouse tissues

Genomic DNA was quantified using DNA standards as described [23]. Reactions were carried out with 600ng of genomic DNA purified using a Qiagen DNAeasy Kit according to the manufacturer’s instructions. The B1 gene of *T*. *gondii* was amplified over 40 cycles using forward 5’-GGAACTGCATCCGTTCATG-3’ and reverse 5’-TCTTTAAAGCGTTCGTGGTC-3’ primer with SyBr Green Master Mix (Life Technologies) and a BioRad CFX real-time thermal cycler. Amplification products were verified using melt-curve analysis.

### Brain IDO gene expression by real-time quantitative PCR analysis

Total RNA was extracted from cerebral cortex and striatum using phenol/chloroform then purified using a column-based method (RNAeasy, Qiagen) with on-column DNAase1 digestion. Gene expression was determined with SYBR Green using intron-spanning primers. IDO1: forward: 5’-CCTTCTGGGAATAAAACACGAGG-3’ and reverse: 5’-CTAAGAAGAAAAGGAAGTTCCGG-3’. IDO2 forward: 5’-ATCTCCACGTAGCTCCTTCT-3’ and reverse: 5’-GCCTCCACACACTGGTTATAG-3’. Expression was normalized to beta-actin using the Applied Biosystems Taqman gene expression primer / probe combination Mm00607939_s1. Gene expression was determined using 17 ng of cDNA per reaction.

### Brain Indoleamine-2,3-dioxygenase activity

Brain IDO activity was determined by HPLC-MS/MS quantification of kynurenine produced after brain protein extract was incubated in excess tryptophan. The method was adapted from previously published protocols [24, 25]. Cerebral cortex and striatum were dissected and snap frozen in dry ice. Brain regions were homogenized in a ten volumes (w/v) of PBS with a power homogenizer for one minute after tissue was visibly dissociated. Tissue homogenate was then sonicated on ice at 70% power for 10 seconds then centrifuged at 9000xg for 5 minutes. Protein was quantified in the supernatant fraction using the Bradford with BSA as standards. The assay incubation buffer [1 mM L-tryptophan, 2 mM methylene blue, 40 mM ascorbic acid, 200 U/ml catalase in 100 mM PBS, pH 6.5] was preheated to 37°C. The brain protein extract was diluted to a concentration of 50 μg in 150 μl of PBS; this was added to 150 μl of preheated incubation buffer at 37°C for 35 minutes. The reaction was stopped by adding 60 μl of 30% (w/v) acetic acid and incubated for 20 minutes at 50°C. Heating the reaction in acidic conditions also hydrolyzes N-formylkynurenine produced by IDO to kynurenine. The mixture was then centrifuged at 12,000xg at 4°C for 5 minutes and the supernatant removed. Sixty microliters of acetonitrile was added then samples were filtered through a Phree Phospholipid Extraction Tube (Phenomenex) and through a 0.4 μm filter before loading into the autosampler for analysis. Kynurenine was detected using a Waters Aquity UPLC-MS/MS with an Acetonitrile/3% Acetic Acid mobile phase gradient on a 2x100 mm Waters BEH C^18^ column. Five μl of sample was injected with a total flow rate of 0.3 ml/min and a total run time of 4.5 minutes. Kynurenine eluted at 1.6 minutes and tryptophan at 2.1 minutes. Both analytes were verified using three M+H parent-daughter transitions. Tryptophan concentrations were measured to verify that a saturating concentration was used for the enzymatic reaction. Kynurenine was quantified using QuanLynx (Waters) software with serial dilutions of kynurenine standards. The limit of quantification for kynurenine using this method was set at 0.01 μM. Assay parameters and specificity were validated *in vivo* using IDO knockout mice (Jackson Labs, Bar Harbor, ME; strain B6.129-*IDO1*^*tm1Alm*^/J), and *in vitro* using the IDO inhibitor 1-methyl-D-tryptophan.

### Lymphocyte phenotypic characterization by flow cytometry

Preparation and analysis of cells was performed similar to that described previously [26, 27]. In brief, mice were sacrificed then immediately perfused with 20 mls of cold 0.9% saline with heparin. Brains were homogenized in 2 mls cold PBS using a Dounce homogenizer. Homogenates were added to 30% percoll® and centrifuged at 2000xg for 20 minutes at 15°C to collect lymphocytes from the pellet. Spleens were crushed through a filter (Falcon) in 2% fetal bovine serum (FBS) and 0.2 mM EDTA in PBS. Erythrocytes were removed using 8.3 g/L ammonium chloride in 0.01M Tris-HCl lysis buffer (pH 7.4). Cells were quantified using a hemocytometer and plated for assays at 1.0 x 10^6^ cells per well. Experimental samples were washed with PBS and incubated on ice for 30 minutes with fixable near-IR Live / Dead stain (Life Technologies). Cells were then incubated with antibodies targeting surface markers and incubated on ice for 30 minutes. Antibodies included Brilliant Violet (BV) 650-conjugated anti-CD4, and BV421-conjugated anti-CD8-alpha (Biolegend). Cells were fixed using the Cytofix/Cytoperm kit with saponin (BD Bioscience). The PE-conjugated anti-Ki67 intracellular antibody was incubated with cells on ice for 45 minutes. Clone number and references for each antibody are provided in S1 Table. Flow cytometry data was collected using Guava an EasyCyte HT flow cytometer (Millipore). Data was analyzed using FlowJo v9.3 software. Cell gating and compensation strategy was determined from no stain, single color, and fluorescence minus one controls for each antibody.

### Functional assessment of CD8+ T-cells *ex vivo*

For assessment of antigen-specific functional response, cells were plated as above in Iscove’s Complete DMEM at 37°C in 5% CO_2_ with 20 μg/ml of Toxoplasma lysate antigen (TLA). Brain cells were plated with 0.5 x 10^6^ congenic marked feeder spleen cells. The TLA was prepared from *T*. *gondii* parasites cultured in fibroblasts. After cell lysis by the parasite, supernatant was filtered through a 3 μm filter, sonicated twice on 70% power for 15 seconds, and stored at -80C in aliquots until use. Parasite protein content was determined by the Bradford assay. After splenic and brain cells were incubated with TLA for 6 hours, 0.65 μl/ml of monensin/brefeldin A cocktail (BD Biosciences) was added to each well for 4 hours to block cytokine secretion. Upon completion, cells were stained with anti-CD4 and anti-CD8 surface antibodies and fixed as above, then stained with PE-conjugated anti-IFNγ intracellular antibody.

### CD8+ T-cell adoptive transfer

Splenocytes were enriched for CD8+ T-cells using EasySep mouse biotin positive selection kit (Stem Cell Technologies) according to the manufacturer’s instructions with biotinylated anti-CD8 (Biolegend, clone 53–6.7 at 2μg/ml). Briefly, spleens from donor mice were removed and crushed through a 70 μm filter and processed as described above. Splenocytes underwent magnetic separation according to manufacturer’s instructions. Isolated CD8+ T-cells were counted and resuspended at 15x10^6^ cells/ml in sterile PBS. Wild-type or HD recipient mice were intravenously injected with 3x10^6^ cells from naïve wild-type or naïve HD mice into the tail vein at 5–6 weeks of age. Mice were then infected by intraperitoneal injection with *T*. *gondii* on the same day.

### Statistical Analyses

All data except survival were analyzed using SAS software version 9.2 (Cary, NC). Generalized linear modeling was used for single time-point analyses and the mixed procedure was used for repeated measures analyses. Assumptions of normality and equal variance were verified for each experiment. All main effect and interactions were investigated in the initial statistical model. When significant effects were present we performed pre-planned pairwise comparisons. Non-normally distributed (IDO enzymatic activity and gene expression) data was log transformed for analysis. These data are presented as means ± 95% confidence intervals as distributions were significantly right skewed. Results for experiments with small group sizes are presented as individual data points. Unless otherwise indicated in the figure legend, these individual data points represent the average of technical triplicates. Survival data was analyzed using Kaplan-Meier analysis with the Holm-Šidák method for pairwise comparisons with Sigma Plot software version 12.5. P-values less than 0.05 were considered significant.

## Results

### Brain IDO activity in HD mice is increased by *T*. *gondii* infection

**Fig 1:**
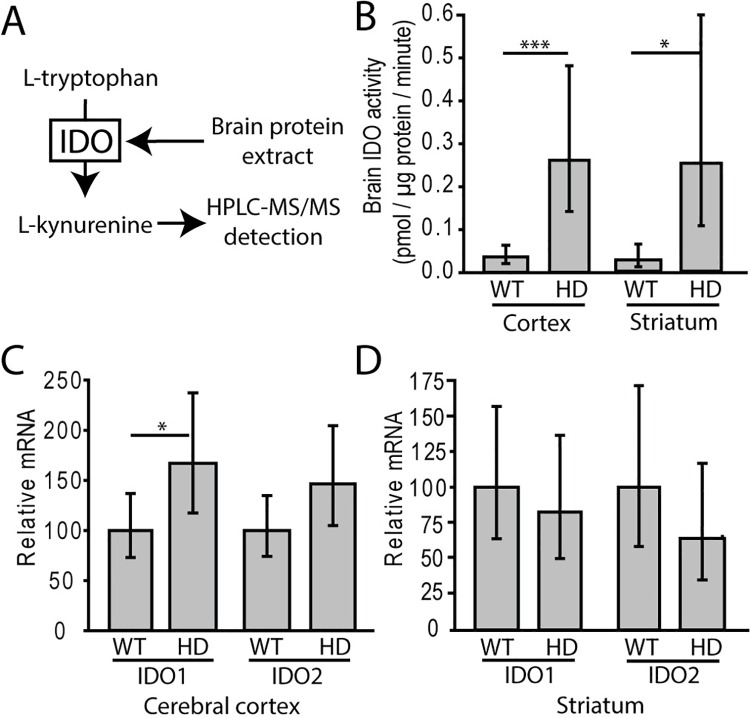
Brain IDO enzymatic activity is increased in early-advanced mouse HD. **A.** Overview of brain IDO assay. Excess tryptophan is added to the brain extract and kynurenine is measured by HPLC-MS/MS. **B.** IDO activity is significantly increased in cerebral cortex and striatum (n = 10 wild-type and 12 HD mice combined from two independent experiments) of HD mice at 14-weeks of age corresponding to early-advanced HD [21]. **C.** Cortical IDO1 (but not IDO2) mRNA transcript is increased in HD mice. **D.** No significant difference was found in IDO1 and IDO2 mRNA levels in striatum of HD mice. **C-D.** Relative expression values were normalized to β-actin. n = 9 wild-type and 10 HD mice combined from two independent experiments. **B-D.** Bars represent means ± 95% confidence intervals. P-values: * = <0.05, *** = <0.001.

**Fig 2:**
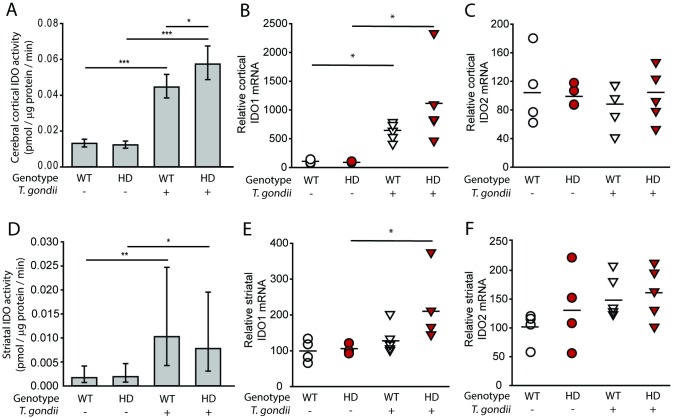
*T*. *gondii*-infected HD mice have elevated brain IDO activity. Wild-type and HD mice were infected around 5-weeks of age and sacrificed 15 days post-infection. **A.** IDO activity is increased in infected WT and HD cerebral cortex. **B-C.** Quantitative PCR for IDO1/2 transcripts in cortex. **B.** Cortical IDO1 transcript is increased in HD and wild-type infected mice. **C.** Cortical IDO2 transcript levels are not altered by HD or *T*. *gondii* infection. **D.** Infection increases IDO activity in striatum of both wild-type and HD mice. **E-F.** Quantitative PCR for IDO1/2 transcripts in striatum. **E.** Striatal IDO1 transcript is increased in HD mice compared to non-infected HD mice. **F.** Striatal IDO2 transcript levels are not altered by HD or *T*. *gondii* infection. **A&D.** Bars represent means ± 95% CI. Data was combined from two independent experiments, n = 9–11 mice. **B-C and E-F.** White circles = wild-type (n = 4), red circles = HD (n = 4), white triangles = wild-type infected (n = 5), red triangles = HD infected (n = 5) p-values: * = <0.05, ** = <0.01, *** = <0.001.

We measured IDO activity (**Fig 1A**) as a marker of kynurenine pathway activity in HD mice. Fourteen-week-old N171-82Q HD mice with early-advanced HD had significant increases in cerebro-cortical and striatal IDO activity compared to wild-type littermates (F_(1,21)_ = 129.01, p<0.0001) (**Fig 1B**). In these mice, mRNA levels of the two IDO isoforms were measured. Cortical IDO1, but not IDO2, was increased in HD mice relative to age-matched controls (**Fig 1C**). No significant increase in striatal mRNA levels of either isoform was detected (**Fig 1D**). To determine if the IDO response to *T*. *gondii* was altered by HD, we infected HD and wild-type mice with *T*. *gondii* and quantified brain IDO activity 15 days later. At this timepoint, HD mice do not show deficits associated with HD. We found that infection significantly elevated IDO activity in cortex (F_(1,34)_ = 49.62, p<0.0001) and striatum (F_(1,39)_ = 12.27, p = 0.0012). Infected HD mice had elevated cortical and striatal IDO activity compared to HD non-infected mice (p<0.0001 and p = 0.0381 respectively) (**Fig 2A & 2D**). Wild-type infected mice had elevated cortical and striatal IDO activity (p<0.0001 and p = 0.0076 respectively) compared with non-infected wild-type mice. Non-infected HD mice did not have increased IDO activity at this time point compared to non-infected wild-type littermates. Interestingly, the increase in cortical (p = 0.0315) but not striatal (p = 0.6718) IDO activity was significantly greater in infected HD compared to infected wild-type mice. Cortical IDO1 mRNA was increased by infection (F_(1,14)_ = 9.06, p = 0.0094); both infected wild-type and HD mice had elevated IDO1 mRNA compared to respective non-infected mice (p = 0.0484 and 0.0123, respectively) (**Fig 2B**). Increases in IDO2 mRNA were absent (**Fig 2C**). Similar effects were seen in the gene expression of IDO1 and IDO2 in striatum with infected HD mice displaying significantly increased IDO1 expression compared to non-infected HD mice (**Fig 2E and 2F**).

### Premature death in *T*. *gondii*-infected HD mice

**Fig 3:**
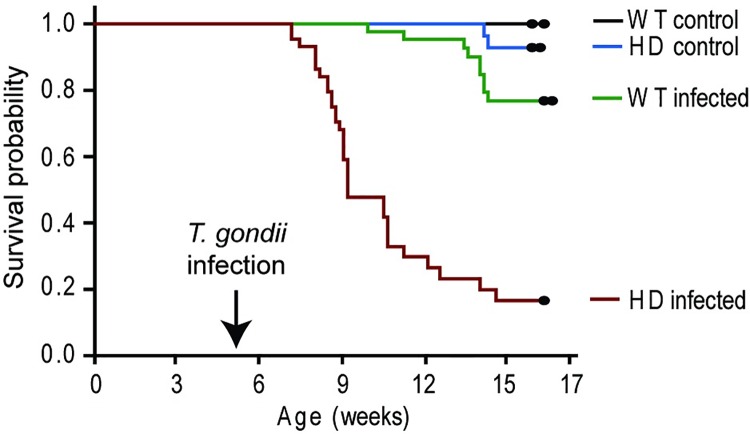
HD mice with *T*. *gondii* infection demonstrate premature death. Mice were infected with 100 *T*. *gondii* bradyzoites by intra-peritoneal injection at 5-weeks of age. **A.** Infected HD mice die significantly earlier than infected wild-type (p = 2.82x10^-10^) and HD non-infected mice (p = 8.014x10^-11^). Wild-type (WT) non-infected: n = 36, HD non-infected: n = 35, WT infected: n = 43, HD infected: n = 44.

To determine if *T*. *gondii* infection affects HD outcomes we infected N171-82Q HD and wild-type mice at 5-weeks of age with the initial goal of determining behavioral outcomes. However, we unexpectedly found that *T*. *gondii* infected HD mice reached euthanasia end points or died spontaneously with losses consistently starting ~2 weeks after infection (p<0.0001) (**Fig 3**). As expected, non-infected HD mice started to die at 14–15 weeks of age. Infected wild-type litter-mate mice had a low rate of mortality beginning about 10 weeks of age (5 weeks post infection) that was significantly greater than non-infected wild-type mice (p = 0.0170). Premature mortality in HD infected mice prevented measurement of behavioral and late-stage HD outcomes.

### *T*. *gondii* infection results in greater parasite burden in HD mice

**Fig 4:**
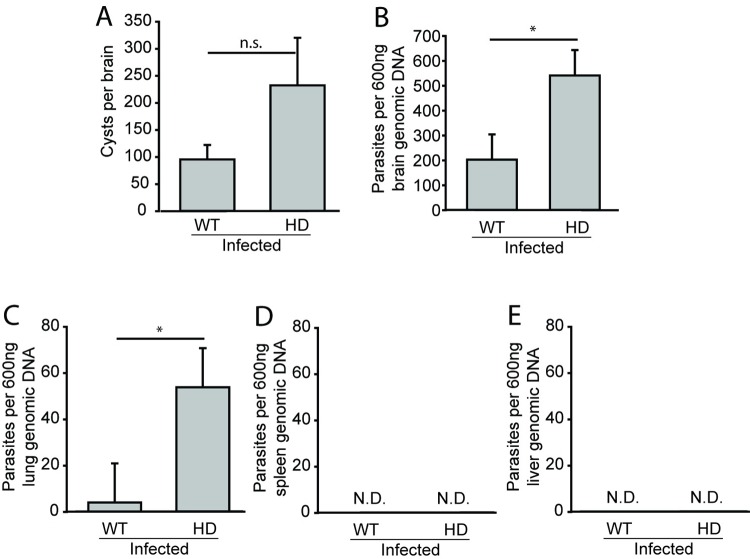
Parasite burden is increased in *T*. *gondii*-infected HD mice. Wild-type and HD mice were infected at 5-weeks of age and sacrificed at 15 dpi. **A.** Brain *T*. *gondii* cyst numbers are low consistent with transition from acute to chronic infection [29]. **B-C.** Parasite burden, assessed by PCR, in brain (B) and lung (C) is greater in infected HD as compared to infected wild-type mice. Parasites were not detected in spleen (**4D**) or liver (**4E**). **A-C.** Bars represent means ± SEM, n = 8 wild-type and n = 9 HD mice combined from two independent experiments. N.D. = not detected, p-values: * = <0.05, ** = <0.01, n.s. = not significant.

The potency of initial *T*. *gondii* immune control impacts tissue burden of *T*. *gondii* parasite stages [28]. To investigate whether HD and wild-type mice immune responses differ following *T*. *gondii* infection, we measured parasite burden as an initial indicator of immune control. Analyses were completed 15 days after infection corresponding to a time point just before onset of mortality in infected HD mice and the same time as increased IDO activity was observed (**Fig 2**). Infected HD mice had a non-significant elevation of parasitic cysts in brain compared to wild-type mice (F_(1,15)_ = 2.46, p = 0.1377) (**Fig 4A**). Quantification of *T*. *gondii* genomic DNA by real-time PCR revealed elevated total parasite load in brain (F_(1,22)_ = 5.52, p = 0.0282) and lung (F_(1,22)_ = 4.73, p = 0.0487) of infected HD mice (**Fig 4B and 4C**). We did not detect parasites in spleen (**4D**) or liver (**4E**) of either infected wild-type or HD groups.

### CD8+ T-cells from *T*. *gondii*-infected HD mice demonstrate reduced proliferation and cellular IFNγ

**Fig 5:**
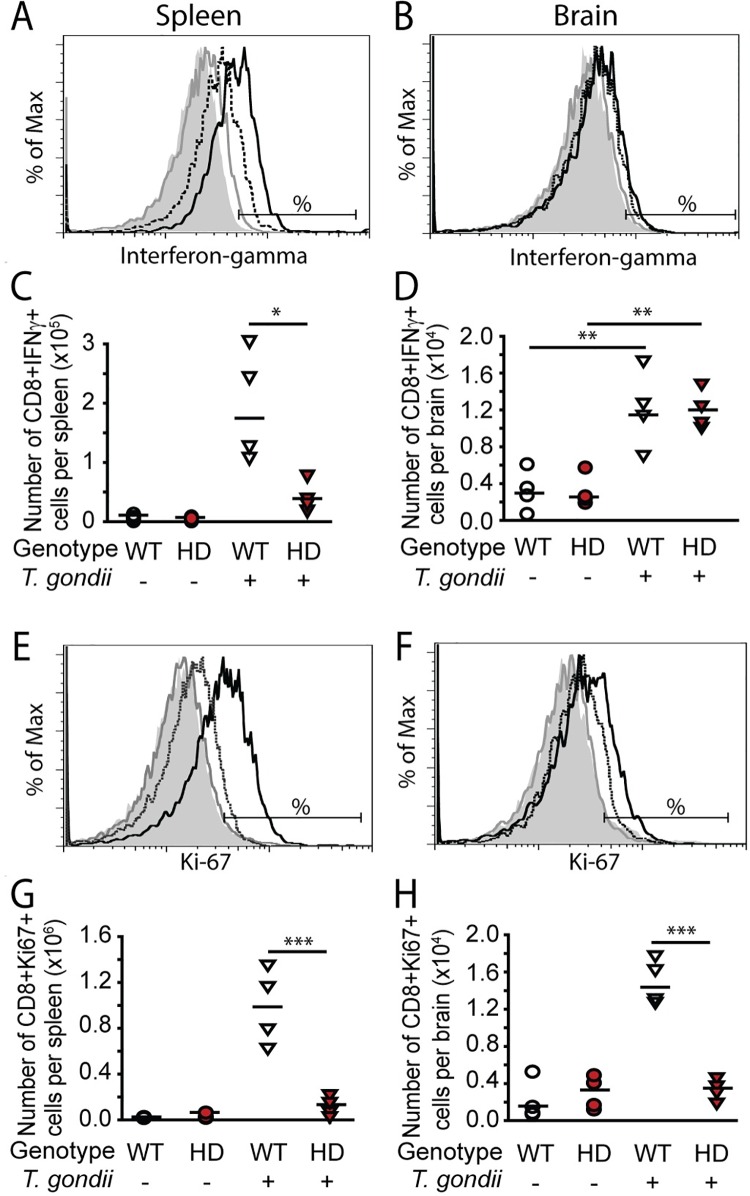
CD8+ T-cell interferon-gamma production and proliferation is decreased in *T*. *gondii*-infected HD mice. HD and wild-type mice were injected ip with 100 *T*. *gondii* bradyzoites or vehicle at 5-weeks of age and sacrificed at 15 dpi. Cells were stimulated *ex-vivo* with *T*. *gondii* lysate to induce an antigen-specific response. Representative fluorescence histogram show IFN-γ production by CD8+ T-cells in spleen (**A**) and brain (**B**) in wild-type non-infected (gray filled), HD non-infected (gray-solid line), wild-type infected (black-solid line), and HD infected (black-dashed line) mice. Horizontal bars show the cutoff for IFN-γ positive cells. **C.** Absolute numbers of splenic CD8+ T-cells producing IFN-γ are decreased in infected HD mice. **D.** Absolute numbers of brain CD8+ T-cells producing IFN-γ are increased in both wild-type and infected HD mice. **E-H.** CD8+ cell proliferation in spleen and brain. Representative fluorescence histograms show relative Ki67 expression in CD8+ T-cells in the spleen (**E**) and brain (**F**). Colored line codes are as described above. Horizontal bars represent the cutoff for Ki67 positive cells. Infected HD mice have significantly decreased absolute numbers of proliferating CD8+ T-cells in spleen (**G**) and brain (**H**) compared to infected wild-type mice. Data points represent the average of technical duplicates from one experiment. White circles = wild-type, red circles = HD, white triangles = wild-type infected, red triangles = HD infected. P-values: * = <0.05, ** = <0.01, *** = <0.001.

CD8+ T-cells are critical for the control of *T*. *gondii* infection; in particular, proliferation and production of IFNγ [14, 26]. Since parasite burdens were increased in HD mice we used flow cytometry to quantify splenic and brain CD8+ T-cells at 15 days post infection. *T*. *gondii* infection resulted in a large increase in splenic (F_(1,12)_ = 36.26, p<0.0001) and brain (F_(1,12)_ = 6.80, p = 0.0229) CD8+ T-cell numbers compared to non-infected controls (**S1 Fig**). To determine if CD8+ T-cells were functioning normally in HD mice, we measured the number of IFNγ+ CD8+ T-cells in HD and wild-type infected mice at 15 days post infection. Infection significantly increased the number of IFNγ+ CD8+ T-cells in both spleen (F_(1,12) =_ 10.01, p = 0.0082) and brain (F_(1,12)_ = 27.90, p = 0.0002). Infected HD mice had significantly fewer IFNγ-producing CD8+ T-cells in the spleen (p = 0.0002), but not brains (p = 0.5802), compared to infected wild-type controls (**Fig 5A–5D**). We also quantified the mean fluorescence intensity (MFI) of IFNγ in CD8+ T-cells to determine relative expression per-cell between HD and wild-type cells. Consistent with the above findings, production of IFNγ was significantly decreased in spleen (p = 0.0007) but not brain (p = 0.7375) of infected HD compared to wild-type infected mice (**S2 Fig**).

In response to infection, antigen specific CD8+ T-cells normally undergo significant expansion which is maintained until infection is controlled [30]. Therefore we measured CD8+ T cell proliferation in infected HD mice. Although both wild-type and HD CD8+ T-cells numbers were similarly increased 15 days post infection (**S1 Fig**), using the cell-cycle progression marker Ki67, we observed a statistically significant interaction of *T*. *gondii* and HD on CD8+ T-cell proliferation in both spleen (F_(2,12)_ = 28.84, p = 0.0002) and brain (F_(2,12)_ = 22.88, p = 0.0004). Infected HD mice had drastically decreased CD8+ proliferation in both spleen (p = 0.0001) and brain (p = 0.0001) as compared to infected wild-type mice (**Fig 5E–5H**). We investigated whether the altered CD8+ cell function might result from an intrinsic defect in these cells resulting from mhtt expression. Naïve wild-type or HD CD8+ T-cells were adoptively transferred into recipient wild-type and HD mice at the time of infection. There was no effect of adoptive transfer of wild-type CD8+ cells in HD mice on survival following *T*. *gondii* infection (**S3 Fig**).

## Discussion

*T*. *gondii* infection is linked with a number of brain disorders. A recent meta-analysis supported an association of infection with schizophrenia, bipolar disorder and obsessive-compulsive disorder [31]. A few studies have additionally addressed a role of latent toxoplasmosis in sporadic neurodegenerative diseases. One study reported sero-positivity rates for *T*. *gondii* in Parkinson’s disease (PD) patients almost twice that of age and sex matched controls [32]. However, another failed to find an association [33]. Serologic-based studies in Alzheimer’s disease (AD) patients have similarly provided both positive and negative findings [34, 35]. Huntington’s disease, unlike PD and AD, is always genetically autosomal dominant with essentially 100% penetrance. Therefore, while *T*. *gondii* does not cause HD it could be an environmental modifier of disease onset and/or progression [5].

Mouse HD models are invaluable for identifying disease-modifying processes and elucidating associated mechanisms. N171-82Q HD mice express an N-terminal fragment of human mhtt and recapitulate many features of human HD [22]. They have been used to study the effects of pharmacologic, nutritional and environmental enrichment on disease outcomes [21, 36, 37]. We report here that these mice have increased IDO activity in brain associated with a shortened life-span and altered CD8 T-cell responses following *T*. *gondii* infection. Prior studies of immune response in HD have demonstrated altered responses to innate challenge by lipopolysaccharide [9]. Studies assessing the effect of immune challenge on adaptive immunity have used *ex-vivo* stimulation and failed to find effects of HD [38]. Here we used *in-vivo* challenge with a live infection to assess the effect on HD mice and infection-associated responses. Importantly, *T*. *gondii* naturally infects mice as well as humans; therefore, we studied a natural infection in this species. Both wild-type and HD mice initially respond normally to infection by increasing CD8+ T-cells in spleen and brain to similar levels (**S1 Fig**). Further, both wild-type and HD mice lacked parasites in spleen and liver at 15 days (**Fig 4D and 4E**) providing additional support for an initial appropriate immune response to infection. However, at this stage of infection, just before mortalities began in HD-infected animals, proliferation of CD8+ T-cells in the brains and spleens of HD-infected compared to wild-type infected mice was decreased (**Fig 5E–5H**). Altered responses of HD monocytes / macrophage to *ex-vivo* challenge are mediated by cell intrinsic effects resulting from mhtt expression [9]. We addressed if early death might be mediated by cell intrinsic deficits in CD8+ T-cells by adoptively transferring wild-type CD8+ T-cells to HD mice. However, there was no effect of adoptive transfer on survival following *T*. *gondii* infection (**S3 Fig**). One caveat to this experiment is that we did not account for the potential of endogenous T-cells to impact the ability of transferred T-cells to respond to infection. Therefore, while the results suggests that dysfunctional CD8+ T-cells responses to infection in HD mice result from cell-extrinsic processes, more studies are needed to investigate the mechanisms involved. Taken together with the small increase in brain parasite load in brain and lung but no detected parasites in spleen or liver (**Fig 4**), findings are consistent with a model in which HD mice initially mount a proper initial CD8+ T-cell response to *T*. *gondii* that then becomes hypo-responsive prior to the onset of mortalities.

Indoleamine-2,3-dioxygenase (IDO) is preferentially expressed in monocyte-derived cells, including, microglia and may have an important role in regulating pathway activity [39]. We show that IDO enzymatic activity is dramatically increased in pathogen-free N171-82Q mouse brain by early-advanced disease (**Fig 1**). These findings are consistent with the work of others demonstrating increased surrogates of IDO activity, namely kynurenine / tryptophan ratios and IDO1 gene upregulation in YAC128 HD mice [40]. Importantly, we found that *T*. *gondii* infection resulted in greater IDO activation in cortex of HD-infected compared to wild-type infected animals (**Fig 2A**). Given that IDO is one marker of KP activity, our findings indicate that chronic *T*. *gondii* infection activates a pathway linked with HD progression. Intriguingly, IDO can also act as a suppressor of T-cell function via promoting kynurenine signaling to the aryl hydrocarbon receptor [41]. While this is one possible mechanism for altered CD8+ T-cell function in HD-infected mice other plausible mechanisms exist including altered antigen presentation and CD4+ T-cell function. More studies are needed to elucidate the complex relationship between IDO, altered CD8+ T-cell function and premature mortality in this mouse HD model.

## Conclusion

We modeled the effect of the prevalent human infection *T*. *gondii* in HD mice and are the first to demonstrate that HD mice have an altered response to a live infectious agent. Findings are relevant to better understanding human HD. While our data supports a role of IDO as a point of intersection in these two diseases the possibility that other mechanisms may be involved cannot be excluded. For example, a recent *in-silico* study reported that *T*. *gondii* interacts with p53 and cholesterol synthesis pathways [42] which are both implicated in HD [43, 44]. One weakness with our study is that the B6/C3H genetic background of the N171-82Q mice used offers moderate susceptibility to infection and prevented the assessment of the effects of a fully latent (clinically silent) infection on HD markers. Future studies will address this by determining the effects of *T*. *gondii* in HD mouse lines with greater background genetic resistance.

## Supporting Information

S1 Fig *T*. *gondii* infection increases CD8+ T-lymphocytes in spleen and brain 15 days post infection. Mice were infected with 100 *T*. *gondii* cyst-free parasites or vehicle intra-peritoneal and sacrificed 15 days later. CD8+ cells were quantified in spleen (**A, C**) and brain (**B, D**). **A-B**. Representative flow cytometry contour plots showing four treatment groups: WT non-infected (top left plot), HD non-infected (top right plot), WT infected (bottom left plot), and HD infected (bottom right plot) from spleen (**A**) and brain (**B**). In each plot, CD4+ T-cells are represented in the top-left quadrant, CD8+ T-cells in the bottom-right quadrant, CD4+CD8+ T-cells in the top-right quadrant, and CD4-CD8- (double-negative cells) in the bottom-left quadrant. **C.** Spleen CD8+ T-lymphocyte absolute numbers increase with infection in WT and HD mice. **D.** Brain CD8+ T-lymphocyte absolute numbers increase in infected WT and HD mice. Data points represent the average of technical duplicates from one experiment. White circles = wild-type (n = 4), red circles = HD (n = 4), white triangles = wild-type infected (n = 4), red triangles = HD infected (n = 4). P-values: * = <0.05, ** = <0.01, *** = <0.001.
(TIF)

S2 Fig HD mice infected with *T*. *gondii* have reduced production of interferon-gamma. The mean fluorescence intensity of interferon-gamma among total CD8+ T-cells is decreased in infected HD mice at 15 days post-infection in spleen (**A**) but not brain (**B**). Data points represent the average of technical duplicates from one experiment. White circles = wild-type, red circles = HD, white triangles = wild-type infected, red triangles = HD infected. P-values: * = <0.05, ** = <0.01, *** = <0.001.
(TIF)

S3 Fig Adoptive transfer of wild-type CD8+ T-cells does not prolong survival of infected HD mice. HD and wild-type mice were infected with *T*. *gondii* at 5-weeks of age. The same day 3x10^6^ CD8+ T-cells from either HD or wild-type mice were adoptively transferred. There is no difference in survival time of HD mice with wild-type donor cells versus HD donor cells. Gray dots represent experiment censoring at 5-weeks post-infection. Red line = HD mice with HD donor cells, green line = HD mice with wild-type donor cells, blue line = wild-type mice with wild-type donor cells, black line = wild-type mice with HD donor cells. n = 10 mice per group.
(TIF)

S1 Table Antibodies used for flow cytometry experiments. All antibodies were obtained from Biolegend. Application references indicate validated, peer-reviewed uses of each antibody clone.
(DOCX)
