# Supplementary figures and images for: Huntingtons Disease Mice Infected with Toxoplasma gondii Demonstrate Early Kynurenine Pathway Activation, Altered CD8+ T-Cell Responses, and Premature Mortality
Source: PLoS One. 2016 Sep 9;11(9):e0162404. doi: 10.1371/journal.pone.0162404 (PMC5017698; doi:10.1371/journal.pone.0162404)

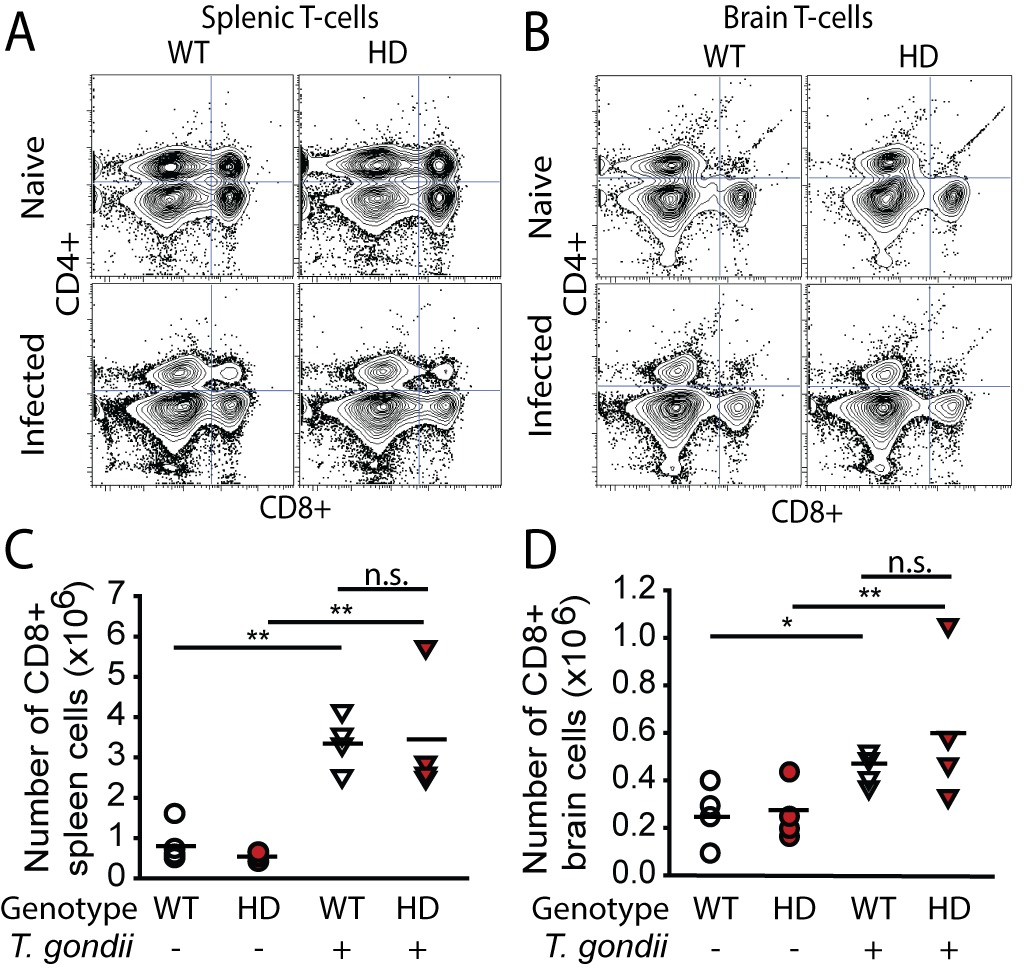

Supplement: S1 Fig — Mice were infected with 100 T. gondii cyst-free parasites or vehicle intra-peritoneal and sacrificed 15 days later. CD8+ cells were quantified in spleen (A, C) and brain (B, D). A-B. Representative flow cytometry contour plots showing four treatment groups: WT non-infected (top left plot), HD non-infected (top right plot), WT infected (bottom left plot), and HD infected (bottom right plot) from spleen (A) and brain (B). In each plot, CD4+ T-cells are represented in the top-left quadrant, CD8+ T-cells in the bottom-right quadrant, CD4+CD8+ T-cells in the top-right quadrant, and CD4-CD8- (double-negative cells) in the bottom-left quadrant. C. Spleen CD8+ T-lymphocyte absolute numbers increase with infection in WT and HD mice. D. Brain CD8+ T-lymphocyte absolute numbers increase in infected WT and HD mice. Data points represent the average of technical duplicates from one experiment. White circles = wild-type (n = 4), red circles = HD (n = 4), white triangles = wild-type infected (n = 4), red triangles = HD infected (n = 4). P-values: * = <0.05, ** = <0.01, *** = <0.001. (TIF) [file pone.0162404.s001.tif]

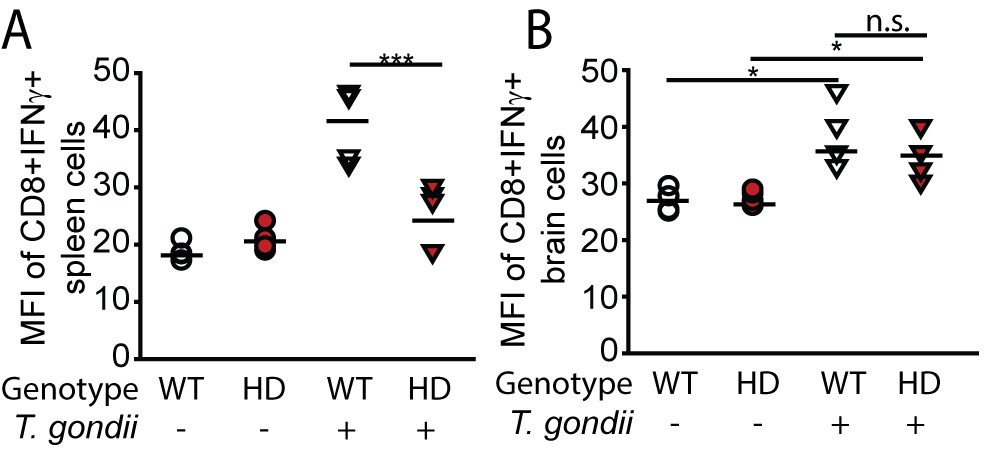

Supplement: S2 Fig — The mean fluorescence intensity of interferon-gamma among total CD8+ T-cells is decreased in infected HD mice at 15 days post-infection in spleen (A) but not brain (B). Data points represent the average of technical duplicates from one experiment. White circles = wild-type, red circles = HD, white triangles = wild-type infected, red triangles = HD infected. P-values: * = <0.05, ** = <0.01, *** = <0.001. (TIF) [file pone.0162404.s002.tif]

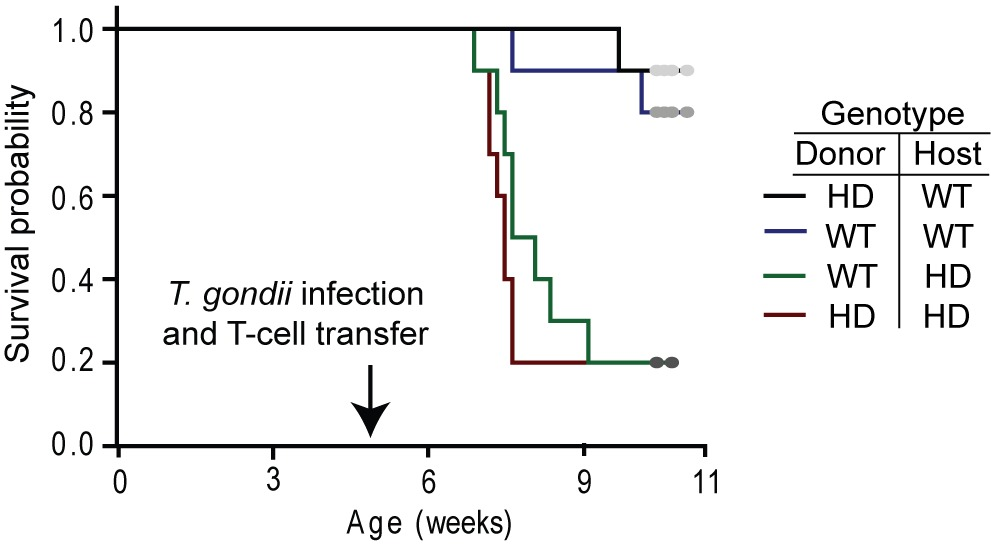

Supplement: S3 Fig — HD and wild-type mice were infected with T. gondii at 5-weeks of age. The same day 3x10^6^ CD8+ T-cells from either HD or wild-type mice were adoptively transferred. There is no difference in survival time of HD mice with wild-type donor cells versus HD donor cells. Gray dots represent experiment censoring at 5-weeks post-infection. Red line = HD mice with HD donor cells, green line = HD mice with wild-type donor cells, blue line = wild-type mice with wild-type donor cells, black line = wild-type mice with HD donor cells. n = 10 mice per group. (TIF) [file pone.0162404.s003.tif]
