# Supplementary material for: Huntingtons Disease Mice Infected with Toxoplasma gondii Demonstrate Early Kynurenine Pathway Activation, Altered CD8+ T-Cell Responses, and Premature Mortality
Source: PLoS One. 2016 Sep 9;11(9):e0162404. doi: 10.1371/journal.pone.0162404 (PMC5017698; doi:10.1371/journal.pone.0162404)
Supplement: S1 Table — All antibodies were obtained from Biolegend. Application references indicate validated, peer-reviewed uses of each antibody clone. (DOCX) [file pone.0162404.s004.docx]

| **Antibody** | **Clone** | **Concentration (μg/10^6^ cells)** | **Reference** |
| --- | --- | --- | --- |
| Anti-CD4 | RM4-5 | 0.2 | [1] |
| Anti-CD8 | 53-6.7 | 0.4 | [2] |
| Anti-Ki67 | 16A8 | 0.4 | [3] |
| Anti-IFN-gamma | XMG1.2 | 0.3 | [4] |
| Anti-CD45.1 | A20 | 0.4 | [5] |

1.	Leon-Ponte M, Ahern GP, O'Connell PJ. Serotonin provides an accessory signal to enhance T-cell activation by signaling through the 5-HT7 receptor. Blood. 2007;109(8):3139-46. doi: 10.1182/blood-2006-10-052787. PubMed PMID: 17158224; PubMed Central PMCID: PMCPMC1852236.

2.	del Rio ML, Cote-Sierra J, Rodriguez-Barbosa JI. Flt3L-mobilized dendritic cells bearing H2-Kbm1 apoptotic cells do not induce cross-tolerance to CD8+ T cells across a class I MHC mismatched barrier. Transpl Int. 2011;24(5):501-13. doi: 10.1111/j.1432-2277.2011.01220.x. PubMed PMID: 21276089.

3.	Sharma SK, Chintala NK, Vadrevu SK, Patel J, Karbowniczek M, Markiewski MM. Pulmonary alveolar macrophages contribute to the premetastatic niche by suppressing antitumor T cell responses in the lungs. Journal of immunology (Baltimore, Md : 1950). 2015;194(11):5529-38. doi: 10.4049/jimmunol.1403215. PubMed PMID: 25911761.

4.	Ko SY, Ko HJ, Chang WS, Park SH, Kweon MN, Kang CY. alpha-Galactosylceramide can act as a nasal vaccine adjuvant inducing protective immune responses against viral infection and tumor. Journal of immunology (Baltimore, Md : 1950). 2005;175(5):3309-17. PubMed PMID: 16116223.

5.	Mende I, Karsunky H, Weissman IL, Engleman EG, Merad M. Flk2+ myeloid progenitors are the main source of Langerhans cells. Blood. 2006;107(4):1383-90. doi: 10.1182/blood-2005-05-1878. PubMed PMID: 16263793; PubMed Central PMCID: PMCPMC1895406.
